# A biochemical method for assessing the neurotoxic effects of misonidazole in the rat.

**DOI:** 10.1038/bjc.1980.337

**Published:** 1980-12

**Authors:** G. P. Rose, A. J. Dewar, I. J. Stratford

## Abstract

A proven biochemical method for assessing chemically induced neurotoxicity has been applied to the study of the toxic effects of misonidazole (MISO) in the rat. This involves the fluorimetric measurement of beta-glucuronidase and beta-galactosidase activities in homogenates of rat nervous tissue. The tissues analysed were sciatic/posterior tibial nerve (SPTN) cut into 4 sections, trigeminal ganglia and cerebellum. MISO administered i.p. to Wistar rats in doses greater than 300 mg/kg/day for 7 consecutive days produced maximal increases in both beta-glucuronidase and beta-galactosidase activities in th SPTN at 4 weeks (140-180% of control values). The highest increases were associated with the most distal secretion of the nerve. Significant enzyme-activity changes were also found in the trigeminal ganglia and cerebellum of MISO-dosed rats. The greatest activity occurred 4-5 weeks after dosing, and was dose-related. It is concluded that, in the rat, MISO can produce biochemical changes consistent with a dying-back peripheral neuropathy, and biochemical changes suggestive of cerebellar damage. This biochemical approach would appear to offer a convenient quantitative method for the detection of neurotoxic effects of other potential radio-sensitizing drugs.


*Br. J. Cancer* (1980) **42**, 890

### A BIOCHEMICAL METHOD FOR ASSESSING THE NEUROTOXIC EFFECTS OF MISONIDAZOLE IN THE RAT

G. P. ROSE, A. J. DEWAR AND I. J. STRATFORD*

*From the Shell Toxicology Laboratory (Tunstall), Sittingbourne Research Centre, Kent ME9 8AG, and the *Institute of Cancer Research, Royal Marsden Hospital, Radiobiology Unit, Physics Department, Sutton, Surrey SM2 5PX*

Received 20 May 1980 Accepted 11 September 1980

**Summary.—A proven biochemical method for assessing chemically induced neurotoxicity has been applied to the study of the toxic effects of misonidazole (MISO) in the rat. This involves the fluorimetric measurement of β-glucuronidase and β-galactosidase activities in homogenates of rat nervous tissue. The tissues analysed were sciatic/posterior tibial nerve (SPTN) cut into 4 sections, trigeminal ganglia and cerebellum. MISO administered i.p. to Wistar rats in doses >300 mg/kg/day for 7 consecutive days produced maximal increases in both β-glucuronidase and β-galactosidase activities in the SPTN at 4 weeks (140–180% of control values). The highest increases were associated with the most distal section of the nerve. Significant enzyme-activity changes were also found in the trigeminal ganglia and cerebellum of MISO-dosed rats. The greatest activity occurred 4–5 weeks after dosing, and was dose-related.**

**It is concluded that, in the rat, MISO can produce biochemical changes consistent with a dying-back peripheral neuropathy, and biochemical changes suggestive of cerebellar damage. This biochemical approach would appear to offer a convenient quantitative method for the detection of neurotoxic effects of other potential radiosensitizing drugs.**

THE 2-NITROIMIDAZOLE, misonidazole (Ro-07-0582, MISO) is at present undergoing clinical trials in many radiotherapy centres as a potential sensitizer of radioresistant hypoxic cells (Dische *et al.*, 1977; Urtasun *et al.*, 1977). However, the clinical effectiveness of MISO and other compounds which might be useful radiosensitizers is limited by neurotoxicity (Dische *et al.*, 1977; Coxon & Pallis, 1976; Le Quesne, 1975). When MISO is given in a fractionated course during radiotherapy, the toxicity first manifests itself as a mild peripheral sensory neuropathy (Dische *et al.*, 1977; Urtasun *et al.*, 1977) and when administration of the drug is continued, some severe convulsions have been observed (Saunders *et al.*, 1978). These contraindications have led to a maximum total drug-dose of 12 g/m$^2$ being adopted (Dische, 1977) the consequence of which is that MISO cannot be used at its optimum radiosensitizing dose. This has led to the search for second-generation hypoxic cell radiosensitizers, based on the nitroimidazole structure, that might be clinically more effective than MISO.

A central requirement for future drug development is an estimation of the probable neurotoxic effect of any promising radiosensitizer. As a first step, the effect of single and multiple doses of MISO on various functional, behavioural and pathological end-points in rodents have been examined. These systems, which include measurement of nerve conduction velocity (Conroy *et al.*, 1979; Hirst *et al.*, 1978, 1979) electron and light microscopy of peripheral nerves and CNS (Adams *et al.*, 1980; Conroy *et al.*, 1979) and behavioural tests such as rota-rod and foot-splay drop-test (Conroy *et al.*, 1979) have shown that

*in vivo* changes can occur after treatment with MISO. However, it is apparent that quantifying such changes in order to identify a superior drug would not be easy.

We report here one way in which a reliable quantitative comparison of the neurotoxicities of different radiosensitizers can be made. In our laboratory we have developed a biochemical method for detecting chemically induced peripheral neuropathies. This method involves measuring the enzymes $\beta$-glucuronidase and $\beta$-galactosidase in homogenates of rat nervous tissue (Dewar & Moffett, 1979). The justification for measuring the activities of these two enzymes is that they have been shown to increase dramatically during the second phase of Wallerian degeneration (Hollinger & Rossiter, 1952; McCaman & Robins, 1959), *i.e.* during the period of proliferation of Schwann cells and macrophages. The majority of chemically induced peripheral neuropathies (with certain exceptions such as lead) are of the primary axonal type and resemble Wallerian degeneration in many respects (Cavanagh, 1973). Therefore, we considered it justifiable to take an increase in the activity of these two enzymes as evidence of degeneration. We have successfully applied this approach to the study of the neurotoxicity of several pesticides and industrial chemicals (Dewar & Moffett, 1979; Dewar *et al.*, 1979).

We report here the results of applying this approach to the study of MISO neurotoxicity. We have examined the effect of subacute administration of MISO on the $\beta$-glucuronidase and $\beta$-galactosidase activities of the sciatic/posterior tibial nerve of the Wistar rat. In view of the evidence that MISO produces a sensory neuropathy in man (Dische *et al.*, 1977; Urtasun *et al.*, 1977) and that nitroimidazole derivatives can produce cerebellar damage in the dog (Scharer, 1972) the trigeminal ganglia and cerebellum were also analysed. Part of this work has been presented as a preliminary communication (Rose *et al.*, 1979).

### METHODS

### *Animals*

Wistar rats bred under specified pathogen-free conditions in the Shell Toxicology Laboratory (Tunstall) Breeding Unit were used throughout. At the start of each of the two experiments the rats were 10–12 weeks old. Before and after dosing they were supplied with food and water *ad libitum.*

### *Dosing*

Misonidazole (Ro-07-0582) (1-(2-nitro-1-imidazolyl)-3-methoxy-2-propanol) was administered as a 5, 10 or 20% w/v solution in propylene glycol (Sigma Chemical Co. Ltd). In Experiment I, 40 male and 40 female Wistar rats received 7 consecutive daily doses of MISO 400 mg/kg/day. A control group of 25 male and 25 female Wistar rats received 7 consecutive daily doses of propylene glycol 2 ml/kg/day. Toxicological signs and body weights were documented throughout the experiment. A high dose of MISO was chosen to ensure that any biochemical correlates of the drug-induced neuropathy would be detectable.

In Experiment II, 5 groups of male Wistar rats (10 per group) received 50, 100, 200, 300 and 500 mg/kg/day MISO for 7 consecutive days. A 6th (control) group received propylene glycol 2 ml/kg/day for 7 consecutive days.

### *Preparation of tissue*

The rats were killed by cervical dislocation. In Expt I, the right and left sciatic nerves were dissected from the spinal cord to the most distal phalangeal branches of the posterior tibial nerve. The length of nerve was then cut into 4 sections ranging from the most distal (Section A) to the most proximal (Section D) as illustrated in Fig. 1. In Expt II, only the two most distal sections of the nerve (A and B) were analysed. In both experiments, the trigeminal ganglia and cerebellum were removed from the skull and cleared of any extraneous tissue. Each tissue sample (with the exception of the cerebellum) was weighed and homogenized in a volume of ice-cold (0–4°C) 0·1M sodium acetate buffer (pH 4·5) containing 0·1% v/v Triton X-100, calculated to yield a 1% w/v homogenate. In the case of the cerebellum a 0·5% w/v homogenate was prepared using the same solutions. These homogenates were used for all subsequent enzyme assays. In Expt I, the enzymes

FIG. 1.—A diagrammatic representation of the sciatic/posterior tibial nerve in a Wistar rat, showing the 4 sections taken for lysosomal enzyme measurements.

β-glucuronidase and β-galactosidase were assayed at 1, 2, 3, 4, 5, 6, 8 and 12 weeks after the start of dosing. In Expt II, the enzymes were assayed after 4 weeks.

*Enzyme assays*

The β-glucuronidase and β-galactosidase activities were measured by methods adapted from those described by Fishman *et al.* (1948), Robins *et al* (1961) and Dewar *et al.* (1975).

(a) *β-Galactosidase.*—A 0·2ml aliquot of each tissue homogenate was diluted with 0·2 ml of 0·1M glycine–HCl buffer (pH 3.0) and added to 1 ml of 1mM methylumbelliferyl galactoside dissolved in 0·1M glycine–HCl buffer (pH 3.0). The buffered substrate had been pre-incubated for 10 min at 37°C in 1·5ml Eppendorf polypropylene tubes. After the contents had been thoroughly mixed, each tube was incubated for 1 h at 37°C in a Techne Dri-Bloc heating unit. The tubes were then centrifuged for 2 min at 10,000 *g* in an Eppendorf microcentrifuge, and the supernatant was decanted into 2 ml of 0·1M $Na_2CO_3$. The fluorescence was measured on a MSE Vitatron (excitation wavelength 365 nm, emission wavelength 450 nm). Standards were prepared by taking 0·01–0·20ml aliquots of a 150μM solution of methylumbelliferone in distilled water and diluting to a final volume of 0·4 ml. This corresponded to a range of 0·26–5·28 μg methylumbelliferone. To each diluted standard was added 1 ml 0·1M glycine–HCl buffer and 2 ml of 0·1M $Na_2CO_3$. The fluorescence was measured as described above. β-Galactosidase activity was expressed as μg methylumbelliferone liberated/h/mg (wet weight).

(b) *β-Glucuronidase.*—A 0·5ml aliquot of each tissue homogenate was placed in a 1·5ml polypropylene tube and diluted with 0·5 ml of 0·1M sodium acetate buffer (pH 4·5). A 0·1ml aliquot of 1mM methylumbelliferyl glucuronide was added, and the contents of each tube were thoroughly mixed, incubated for 2 h at 37°C and then centrifuged for 2 min at 10,000 *g*. The supernatant was then decanted into 2·3 ml 0·1M $Na_2CO_3$ and the fluorescence was measured as for β-galactosidase. The methylumbelliferone liberated was calculated by reference to the methylumbelliferone standard curve, the preparation of which is described under (a). The β-glucuronidase activity was expressed as μg methylumbelliferone liberated/h/mg (wet weight). The liberation of the fluorescent product methylumbelliferone has been shown in our laboratory (Rose & Dewar, unpublished) and by McCaman & Robins (1959) to be linear for at least 10 h. The substrates used also gave negligible fluorescence under the above conditions, and non-enzymatic hydrolysis was not detected by the above method.

### RESULTS

Rats dosed with 400 mg/kg/day MISO became progressively more lethargic. This was accompanied by a reduction in the rate of respiration, hypothermia and cyanosis of the limb extremities, tail and scrotum. After 5 doses (a cumulative dose of 2 g/kg) the rats showed reduced muscle tone, hunched hindquarters and an abnormal (tip-toe) hindlimb gait. These signs were seen in most of the animals, and lasted for up to 3 weeks from the start of dosing. Twelve of the animals died, mostly in the first week after dosing.

β-Glucuronidase and β-galactosidase activities in the sciatic/posterior tibial nerves (SPTN) are shown in Figs 2 and 3. The MISO-dosed animals showed greater nerve β-glucuronidase and β-galactosidase activities than the propylene glycol-dosed controls. These increases were maximal

TABLE I.—*Effect of increasing doses of misonidazole on the β-glucuronidase and β-galactosidase activities in distal sections of the sciatic/posterior tibial nerve of male Wistar rats*

| MISO administered per day (mg/kg) | Cumulative dose over 7 days (mg/kg) | Enzyme activity expressed as μg of methylumbelliferone liberated/h/mg (wet weight) (means ± s.e.) — Section A: β-Glucuronidase | Section A: β-Galactosidase | Section B: β-Glucuronidase | Section B: β-Galactosidase |
|---|---|---|---|---|---|
| — | 0 | 0·34 ± 0·01 (20) | 1·39 ± 0·05 (20) | 0·25 ± 0·01 (20) | 1·08 ± 0·03 (20) |
| 50 | 350 | 0·33 ± 0·01 [96·2%] (20) | 1·40 ± 0·04 [100·6%] (20) | 0·26 ± 0·01 [102·0%] (20) | 1·12 ± 0·03 [103·9%] (20) |
| 100 | 700 | 0·34 ± 0·01 [100·0%] (22) | 1·51 ± 0·04 [108·4%] (22] | 0·25 ± 0·01 [100·0%] (22) | 1·17 ± 0·04 [107·8%] (22) |
| 200 | 1400 | 0·37 ± 0·01 [107·9%] (14) | 1·49 ± 0·06 [107·0%] (14) | 0·27 ± 0·01 [106·3%] (14) | 1·17 ± 0·05 [108·2%] (14) |
| 300 | 2100 | 0·40 ± 0·01** [118·1%] (14) | 1·68 ± 0·05** [120·6%] (14) | 0·28 ± 0·01** [111·5%] (14) | 1·22 ± 0·04* [113·0%] (14) |
| 500 | 3500 | 0·53 ± 0·04*** [156·3%] (6) | 1·87 ± 0·11*** [134·5%] (6) | 0·32 ± 0·01*** [124·5%] (6) | 1·31 ± 0·04*** [121·3%] (6) |

Significant difference from control: *$P < 0·05$, **$P < 0·01$, ***$P < 0·001$.
Enzyme activity measured 4 weeks after the start of dosing.

TABLE II.—*Effect of the subacute administration of MISO on the β-glucuronidase and β-galactosidase activities in the trigeminal ganglia of male and female Wistar rats*

| Time of analysis (weeks) | Enzyme activity expressed as μg of methylumbelliferone liberated/h/mg (wet weight) — β-Glucuronidase: Propylene glycol 2 ml/kg/day | β-Glucuronidase: MISO 400 mg/kg/day | β-Galactosidase: Propylene glycol 2 ml/kg/day | β-Galactosidase: MISO 400 mg/kg/day |
|---|---|---|---|---|
| 1 | 0·31 ± 0·01 (12) | 0·35 ± 0·01*** [113·3%] (18) | 1·12 ± 0·02 (12) | 1·23 ± 0·02*** [109·5%] (18) |
| 2 | 0·24 ± 0·02 (10) | 0·21 ± 0·01** [86·4%] (18 | 0·95 ± 0·03 (10) | 1·04 ± 0·03 [109·4%] (18) |
| 3 | 0·19 ± 0·01 (14) | 0·21 ± 0·01 [106·8%] (18) | 0·93 ± 0·05 (14) | 0·98 ± 0·03 [105·2%] (18) |
| 4 | 0·19 ± 0·01 (10) | 0·28 ± 0·02** [145·9%] (10) | 1·02 ± 0·04 (10) | 1·25 ± 0·06** [122·0%] (10) |
| 5 | 0·21 ± 0·01 (12) | 0·31 ± 0·01*** [151·7%] (12) | 1·08 ± 0·06 (12) | 1·35 ± 0·08* [124·8%] (12) |
| 6 | 0·23 ± 0·01 (6) | 0·31 ± 0·01*** [135·4%] (8) | 1·03 ± 0·04 (6) | 1·19 ± 0·03** [115·4%] (8) |
| 8 | 0·21 ± 0·01 (12) | 0·24 ± 0·01*** [118·0%] (14) | 1·05 ± 0·02 (12) | 1·15 ± 0·02* [109·7%] (14) |
| 12 | 0·22 ± 0·01 (12) | 0·25 ± 0·01* [111·2%] (10) | 1·25 ± 0·06 (12) | 1·23 ± 0·09 [101·1%] (10) |

Significance difference from control: *$P < 0·05$, **$P < 0·01$, ***$P < 0·001$.

FIG. 2.—Effect of subacute administration of MISO on the β-glucuronidase activity in 4 sections of the sciatic/posterior tibial nerves from male and female Wistar rats.

| Sampling time in weeks | 1 | 2 | 3 | 4 | 5 | 6 | 8 | 12 |
|---|---|---|---|---|---|---|---|---|
| Number of determinations at each sampling time | | | | | | | | |
| MISO | 18 | 18 | 18 | 20 | 12 | 8 | 14 | 10 |
| Control | 12 | 10 | 14 | 16 | 12 | 6 | 12 | 12 |

I——I 7 consecutive daily doses; ▲——▲ MISO 7 × 400 mg/kg/day; ●——● Propylene glycol 7 × 2 ml/kg/day.

Significant difference from mean control value: $^{*}P < 0{\cdot}05$, $^{**}P < 0{\cdot}01$, $^{***}P < 0{\cdot}001$.

FIG. 3.—Effect of subacute administration of MISO on the β-galactosidase activity in 4 sections of the sciatic/posterior tibial nerves from male and female Wistar rats. Symbols as in Fig. 2.

4 weeks after the start of dosing. By 12 weeks the enzyme activities had returned to within the control range. The largest and most significant increases were seen in the most distal section (A). Progressively smaller increases were found in sections (B), (C) and (D); the enzyme changes in (D) were not statistically significant. On the basis of these findings (Expt. I) enzyme activities in the 2 most distal sections of the nerve (A and B) were then analysed 4 weeks after the administra-

TABLE III.—*Effect of increasing doses of MISO on the β-glucuronidase and β-galactosidase activities in the trigeminal ganglia of male Wistar rats*

| MISO adminis-tered per day (mg/kg) | Cumulative dose over 7 days (mg/kg) | Enzyme activity expressed as μg of methylumbelliferone liberated/h/mg (wet weight) β-Glucuron-idase | β-Galactos-idase |
|---|---|---|---|
| | 0 | 0·32 ± 0·01 (20) | 1·29 ± 0·05 (20) |
| 50 | 350 | 0·34 ± 0·01 [106·3%] (20) | 1·36 ± 0·05 [105·1%] (20) |
| 100 | 700 | 0·35 ± 0·01* [111·4%] (22) | 1·46 ± 0·05* [112·9%] (22) |
| 200 | 1400 | 0·38 ± 0·01** [119·6%] (14) | 1·45 ± 0·06 [112·6%] (14) |
| 300 | 2100 | 0·42 ± 0·01*** [133·2%] (14) | 1·61 ± 0·03** [124·4%] (14) |
| 500 | 3500 | 0·47 ± 0·03*** [148·1%] (6) | 1·72 ± 0·05*** [133·3%] (6) |

Significant difference from control: *$P < 0·05$, **$P < 0·01$, ***$P < 0·001$.

Enzyme activity measured 4 weeks after the start of dosing.

TABLE V.—*Effect of increasing doses of MISO on the β-glucuronidase and β-galactosidase activities in the cerebellum of male Wistar rats*

| MISO per day (mg/kg) | Cumulative dose over 7 days (mg/kg) | Enzyme activity expressed as μg of methylumbelliferone liberated/h/mg (wet weight) β-Glucuron-idase | β-Galactos-idase |
|---|---|---|---|
| | 0 | 0·89 ± 0·02 (10) | 2·16 ± 0·06 (10) |
| 50 | 350 | 0·87 x 0·02 [97·9%] (10 | 1·96 x 0·05* [90·7%] (10) |
| 100 | 700 | 0·89 ± 0·02 [100·6%] (11) | 1·80 ± 0·03** [87·0%] (11) |
| 200 | 1400 | 0·96 ± 0·03 [108·2%] (7) | 1·8 ± 20·04** [84·4%] (7) |
| 300 | 2100 | 1·02 ± 0·03** [114·8%] (7) | 1·71 ± 0·05*** [79·1%] (7) |
| 500 | 3500 | 1·11 ± 0·07*** [125·1%] (3) | 1·49 ± 0·07*** [69·3%] (3) |

Significant difference from control: *$P < 0·05$, **$P < 0·01$, ***$P < 0·001$.

Enzyme activity measured 4 weeks after the start of dosing.

tion of MISO at doses ranging from 50 to 500 mg/kg/day (Expt II). The results in Table I indicate that the MISO-induced increases in β-glucuronidase and β-galactosidase activities were dose-related.

Significant increases in the activities of β-glucuronidase and β-galactosidase were also found in the trigeminal ganglia of MISO-dosed animals. These activity increases were maximal at 4–5 weeks (Table II) and dose-related (Table III). A dose of 200/mg/kg/day MISO was required to produce a statistically significant increase.

The β-glucuronidase activity in the cerebellum increased significantly after MISO dosage. The increase was maximal at 4 weeks (Table IV) and dose-related (Table V). In contrast, the β-galactosidase activity in the cerebellum decreased after MISO, reaching a minimum at 5 weeks (Table V). This decrease was also dose-related (Table V).

### DISCUSSION

Subacute administration of high doses of MISO elicited biochemical changes in the sciatic/posterior tibial nerve (SPTN) that were qualitatively similar to those observed in Wallerian degeneration (Hollinger & Rossiter, 1952; McCaman & Robins, 1959) and chemically induced peripheral neuropathies (Dewar & Moffett, 1979; Dewar *et al.*, 1979). In chemically induced neuropathies of the dying-back type (*e.g.* that produced by acrylamide) the largest increases in β-glucuronidase activity are found in the distal portion of the nerve, with minimal changes in the proximal portion (Dewar & Moffett, 1979). In contrast, in the neuropathy produced by methyl mercury, the enzyme activity increases appear contemporaneously along the whole length of the nerve (Dewar & Moffett, 1979). Thus, it may be concluded that the enzyme activity increases found

TABLE IV.—*Effect of the subacute administration of MISO on the β-glucuronidase and β-galactosidase activities in the cerebellum of male and female Wistar rats*

Enzyme activity expressed as μg of methylumbelliferone liberated/h/mg (wet weight)

| Time of analysis (weeks) | β-Glucuronidase Propylene glycol 2 ml/kg/day | β-Glucuronidase MISO 400 mg/kg/day | β-Galactosidase Propylene glycol 2 ml/kg/day | β-Galactosidase MISO 400 mg/kg/day |
|---|---|---|---|---|
| 1 | 0·39 ± 0·02 (6) | 0·47 ± 0·01** [118·9%] (9) | 1·78 ± 0·04 (6) | 1·34 ± 0·05*** [75·1%] (9) |
| 2 | 0·33 ± 0·01 (5) | 0·45 ± 0·02*** [137·2%] (9) | 2·05 ± 0·18 (5) | 1·66 ± 0·09** [80·7%] (9) |
| 3 | 0·33 ± 0·01 (14) | 0·49 ± 0·01*** [147·6%] (18) | 2·18 ± 0·05 (14) | 1·55 ± 0·05*** [70·9%] (18) |
| 4 | 0·40 ± 0·03 (8) | 0·83 ± 0·04*** [205·5%] (10) | 2·01 ± 0·06 (8) | 1·45 ± 0·06*** [71·9%] (10) |
| 5 | 0·31 ± 0·02 (6) | 0·44 ± 0·03** [142·1%] (6) | 1·95 ± 0·10 (6) | 1·36 ± 0·08** [69·8%] (6) |
| 6 | 0·32 ± 0·01 (3) | 0·42 ± 0·01** [132·9%] (4) | 1·80 ± 0·08 (3) | 1·18 ± 0·03** [65·8%] (4) |
| 8 | 0·54 ± 0·02 (6) | 0·54 ± 0·02 [106·0%] (7) | 2·32 ± 0·10 (6) | 2·21 ± 0·09 [95·1%] (7) |
| 12 | 0·59 ± 0·02 (6) | 0·57 ± 0·01 [97·3%] (5) | 2·61 ± 0·1 (6) | 2·62 ± 0·14 [100·7%] (5) |

Significant difference from control: $^{**}P < 0·01$, $^{***}P < 0·001$.

in the SPTN of MISO-dosed rats are consistent with a dying-back neuropathy.

On the basis of the data in Table I a cumulative dose of 2·1 g or more is required to produce biochemical changes consistent with peripheral neuropathy. However, even at doses of MISO sufficient to kill a proportion of the animals, the mean increases in β-glucuronidase and β-galactosidase in the most affected part of the nerve were relatively small (≤80%; Table I, Figs 2 and 3). This is considerably less than the increases of 300–600% that have been found after neurotoxic doses of acrylamide or methyl mercury (Kaplan & Murphy, 1972; Dewar & Moffett, 1979). This suggests that the peripheral nerve damage produced by MISO, even at near lethal doses, is small compared with that produced by neurotoxic doses of acrylamide and methyl mercury.

Methyl mercury, a compound which produces sensory neuropathy in rats, has been shown to increase β-glucuronidase and β-galactosidase activities in the trigeminal ganglia (Dewar & Moffett, 1979). Qualitatively similar (*i.e.* in time course) but smaller changes were found in the trigeminal ganglia of MISO-treated rats. This suggests degeneration in the trigeminal ganglia, or possibly a chromatolytic response in the neurones of the ganglia to degeneration in the axons of the trigeminal nerve. In either case the biochemical evidence suggests that MISO affects sensory nerve fibres. This is consistent with MISO-induced neuropathy in humans, which is believed to be primarily sensory (Dische *et al.*, 1977; Urtasun *et al.*, 1977).

There is evidence that certain types of degeneration in the CNS are accompanied by large increases in $\beta$-glucuronidase activity. This is particularly true of degeneration in which cellular proliferation is a feature, *e.g.* the encephalopathy produced by the copper-chelating agent cuprizone (Bowen *et al.*, 1974). There is also evidence that $\beta$-galactosidase can be regarded as a neuronal marker in the CNS (Sinha & Rose, 1972) and that neuronal damage or loss is accompanied by a reduction in the activity of this enzyme, *e.g.* in the encephalopathy produced by Semliki Forest virus (Bowen *et al.*, 1974). On the basis of this evidence the results shown in Tables IV and V suggest that MISO can induce degenerative changes in the rat cerebellum.

Shortly after this biochemical study was completed, a detailed neuropathological examination of the effects of MISO on rats was published (Griffin *et al.*, 1979). The results of that study were consistent with the biochemical findings reported here, in that there was evidence of a mild dying-back peripheral neuropathy. Furthermore, necrosis and degeneration of the cerebellar-roof nuclei was reported. Neuropathological evidence of damage to cerebellar Purkinje cells has been found in dogs dosed with nitroimidazole derivatives (Scharer, 1972).

In summary, we have obtained biochemical evidence to suggest that misonidazole, when administered in large doses to rats, produces a mild dying-back neuropathy, which is consistent with available neuropathological evidence. Since this biochemical method of detecting chemically induced neuropathy is technically simple and can be performed rapidly on a large number of nerve samples, it would appear to offer a simple sensitive method for screening other candidate radiosensitizing drugs for neurotoxic effects. The biochemical technique has the advantage that it yields data in a quantitative form which can be used for the construction of dose–response graphs, thus enabling convenient comparison between the neurotoxic effects of different drugs. In our experience (Rose & Dewar, 1980) alternative methods, such as behavioural or functional tests are ineffective in detecting the neurotoxic effects of MISO reliably. A similar biochemical approach could be used for screening for possible neurotoxic effects in the cerebellum, but here the correlation between the biochemical findings and the neuropathy is less defined and may require further investigation.

### REFERENCES

Bowen, D. M., Flack, R. H. A., Martin, R. O., Smith, C. B., White, P. & Davison, A. N. (1974) Biochemical studies on degenerative neurological disorders, I: Acute experimental encephalitis. *J. Neurochem.*, **22**, 1099.

Cavanagh, J. B. (1973) Peripheral neuropathy caused by chemical agents. *CRC Crit. Rev. Toxicol.*, **2**, 365.

Conroy, P. J., von Burg, R., Passalacqua, W., Penney, D. P. & Sutherland, R. M. (1979) Misonidazole neurotoxicity in the mouse. *Int. J. Rad. Onc. Biol. Phys.*, **5**, 983.

Coxon, A. & Pallis, C. A. (1976) Metronidazole neuropathy. *J. Neurol. Neurosurg. Psychiat.*, **39**, 403.

Dewar, A. J., Barron, G. & Reading, H. W. (1975) The effect of retinol and acetylsalicyclic acid on the release of lysosomal enzymes from the rat retina *in vitro*. *Exp. Eye Res.*, **20**, 63.

Dewar, A. J. & Moffet, B. J. (1979) Biochemical methods for detecting neurotoxicity—a short review. In *Pharmacological Methods in Toxicology*. Ed. Zbinden and Gross. Oxford: Pergamon Press. p. 545.

Dewar, A. J., Moffet, B. J. & Rose, G. P. (1979) A biochemical approach to neurotoxicity testing. XI *Int. Cong. Biochem.* Toronto, Canada: NRCC. p. 550.

Dische, S. (1977) Radiosensitizers in the treatment of squamous cell carcinoma of head and neck. *Clin. Otolaryngol.*, **2**, 403.

Dische, S., Saunders, M. I., Lee, M. E., Adams, G. E. & Flockhart, I. R. (1977) Clinical testing of the radiosensitizers RO–07–0582: Experience with multiple doses. *Br. J. Cancer*, **35**, 567.

Fishman, W. H., Springer, B. & Brunetti, P. (1948) Application of an improved glucuronidase assay method to the study of human blood $\beta$-glucuronidase. *J. Biol. Chem.*, **173**, 449.

Griffin, J. W., Price, D. L., Knethe, O. D. & Goldberg, A. M. (1979) Neurotoxicity of misonidazole in rats. I. Neuropathology. *Neurotoxicology*, **1**, 299.

Hirst, D. E., Vojnovic, B., Stratford, I. J. & Travis, E. L. (1978) The effect of the radiosensitiser misonidazole on motor nerve conduction velocity in the mouse. *Br. J. Cancer*, **37** (Suppl. III), 237.

Hirst, D. G., Vojnovic, B. & Hobson, B. (1979) Changes in nerve conduction velocity in the mouse after acute and chronic administration of nitroimidazoles. *Br. J. Cancer*, **39**, 159.

Hollinger, D. M. & Rossiter, R. J. (1952) Chemical studies of peripheral nerve during Wallerian degeneration, V: β-glucuronidase. *Biochem. J.*, **52**, 659.

KAPLAN, M. L. & MURPHY, S. D. (1972) Effect of acrylamide on rota-rod performance and sciatic nerve β-glucuronidase activity of rats. *Toxicol. Appl. Pharmacol.*, **22**, 259.

LEQUESNE, P. M. (1975) Neuropathy due to drugs. In *Peripheral Neuropathy*. Ed. Dyke *et al.* Philadelphia: Saunders. p. 1263.

MCCAMAN, R. E. & ROBINS, E. (1959) Quantitative biochemical studies of Wallerian degeneration in the peripheral and central nervous system, II: Twelve enzymes. *J. Neurochem.*, **5**, 32.

ROBINS, E., FISHER, K. & LOWE, I. P. (1961) Quantitative histochemical studies of the morphogenesis of the cerebellum. *J. Neurochem.*, **8**, 96.

ROSE, G. P., DEWAR, A. J. & STRATFORD, I. J. (1979) A biochemical assessment of the neurotoxicity of the radiosensitizing drug misonidazole, in the rat. *1st Int. Cong. Neurotoxicity* (Varese, Italy), p. 158.

ROSE, G. P. & DEWAR, A. J. (1980) A biochemical and function appraisal of misonidazole-induced neurotoxicity, in the rat. *Workshop on Neurotoxic Properties of Misonidazole and Other Radiosensitizers.* Ludwig Inst., Sutton, U.K.

SAUNDERS, M. I., DISHE, S., ANDERSON, P. & FLOCKHART, I. R. (1978) The neurotoxicity of misonidazole and its relationship to dose, half-life and concentration in serum. *Br. J. Cancer*, **37** (Suppl. III), 268.

SCHARER, K. (1972) Selective alterations of Purkinje cells in the dog after oral administration of high doses of nitroimidazole derivatives. *Verh. Dtsch Ges. Pathol.*, **56**, 407.

SINHA, A. K. & ROSE, S. P. R. (1972) Compartmentation of lysosomes in neurons and neuropil, and a new neuronal marker. *Brain Res.*, **39**, 181.

URTASUN, R. C., BAND, P. R., CHAPMAN, J. D., RABIN, H., WILSON, A. F. & FRYER, C. G. (1977) Clinical phase I study of the hypoxic cell radiosensitizer Ro-07-0582, a 2-nitro-imidazole derivative. *Radiology*, **122**, 801.


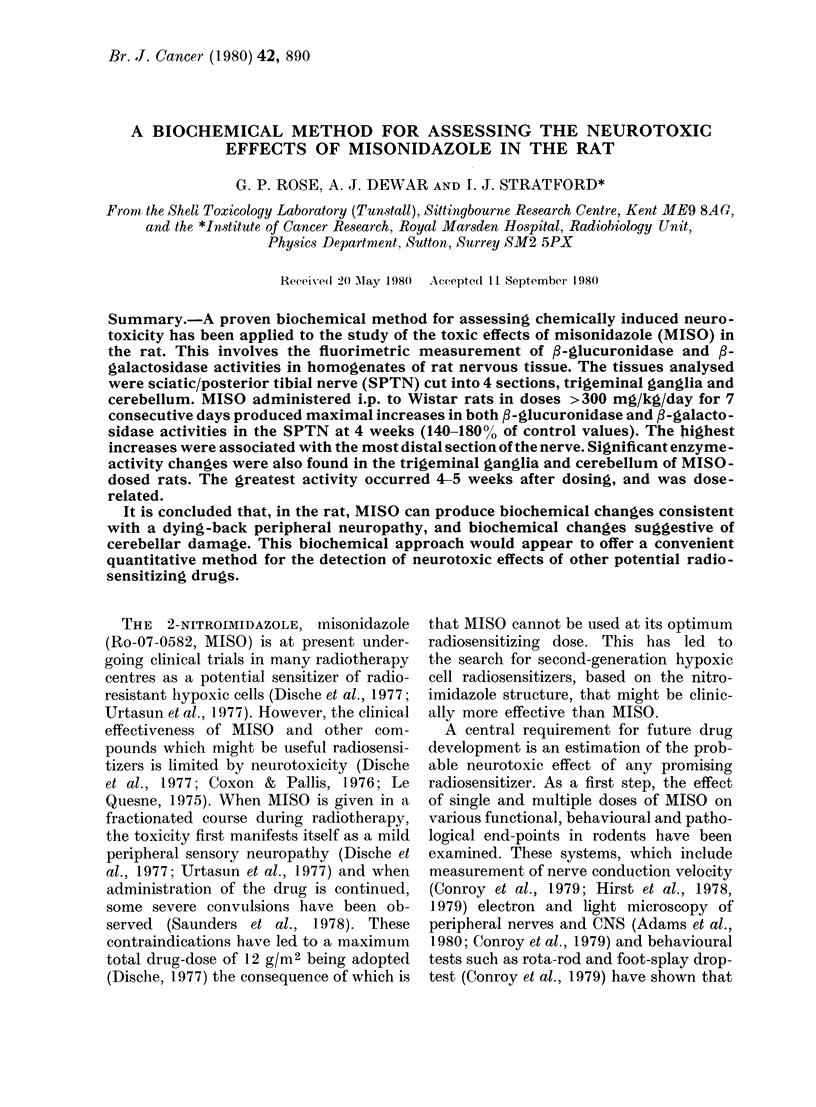

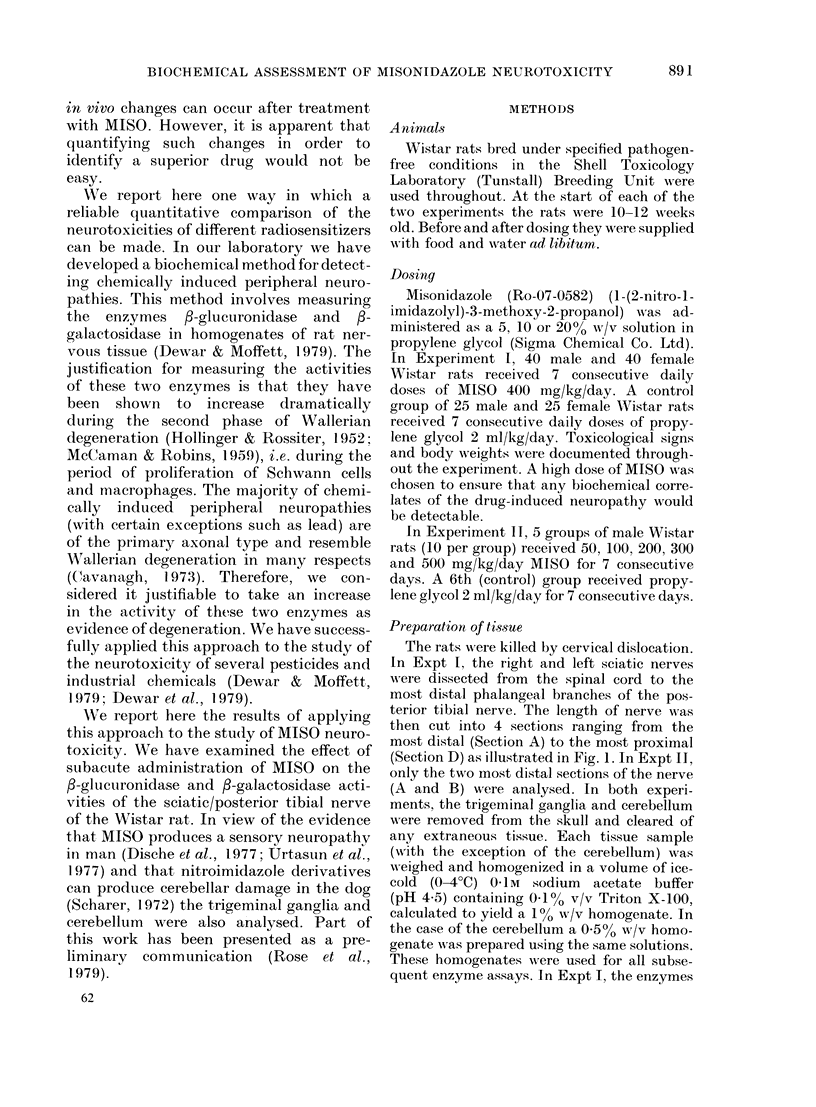

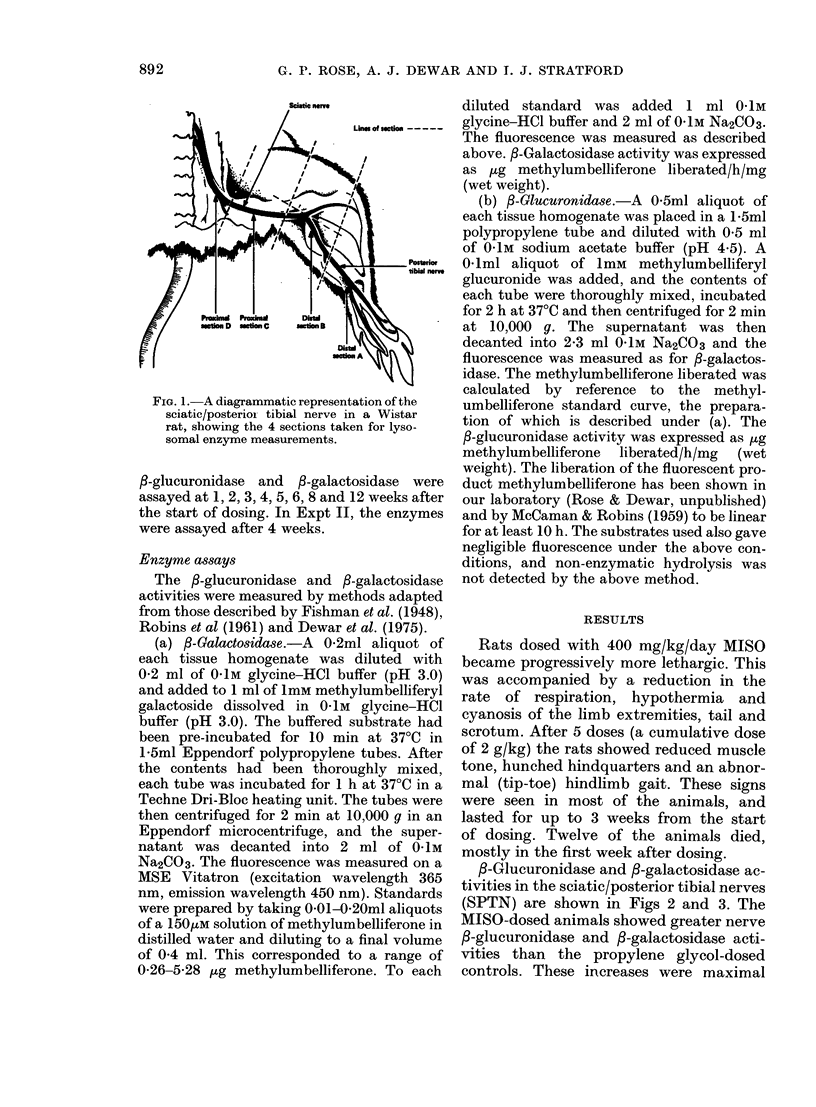

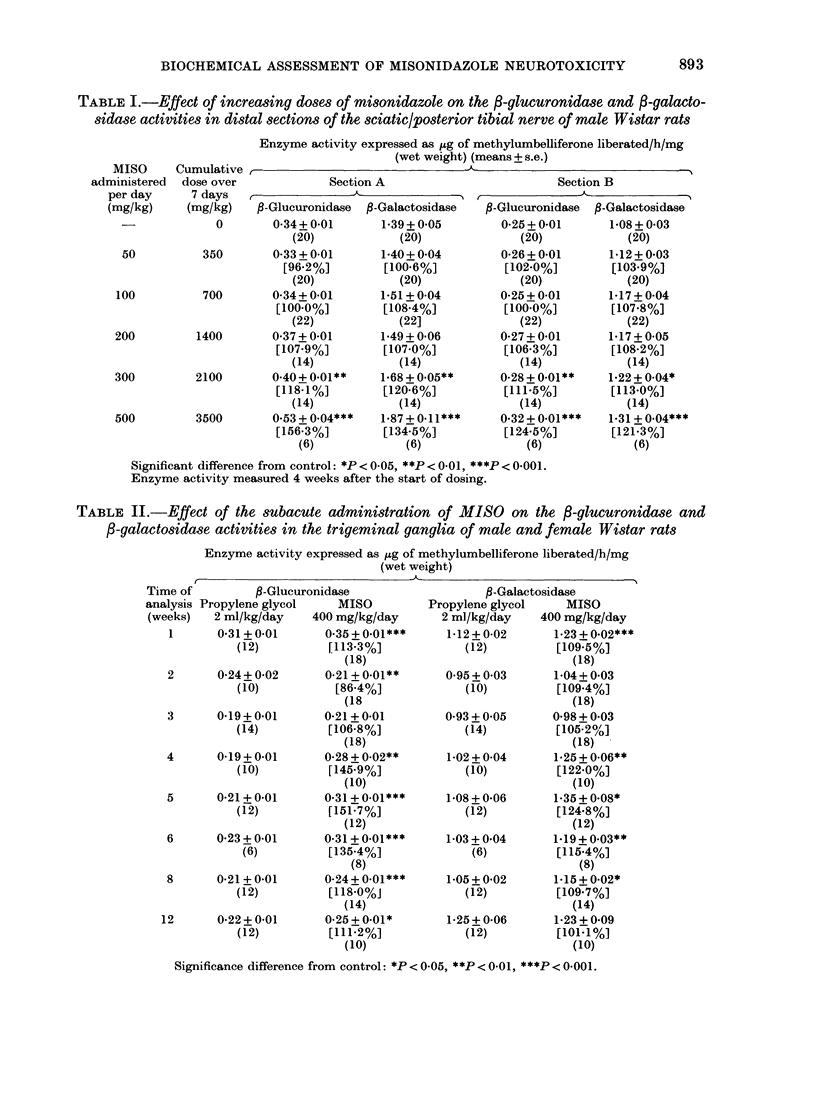

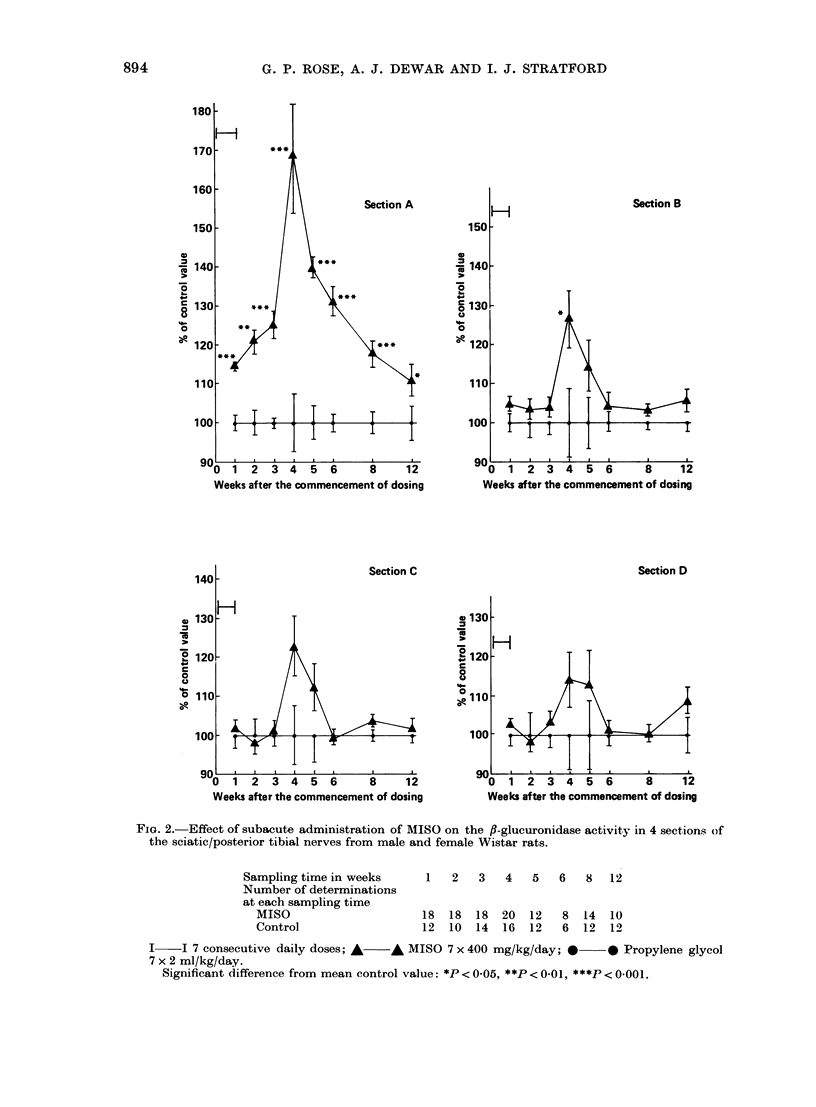

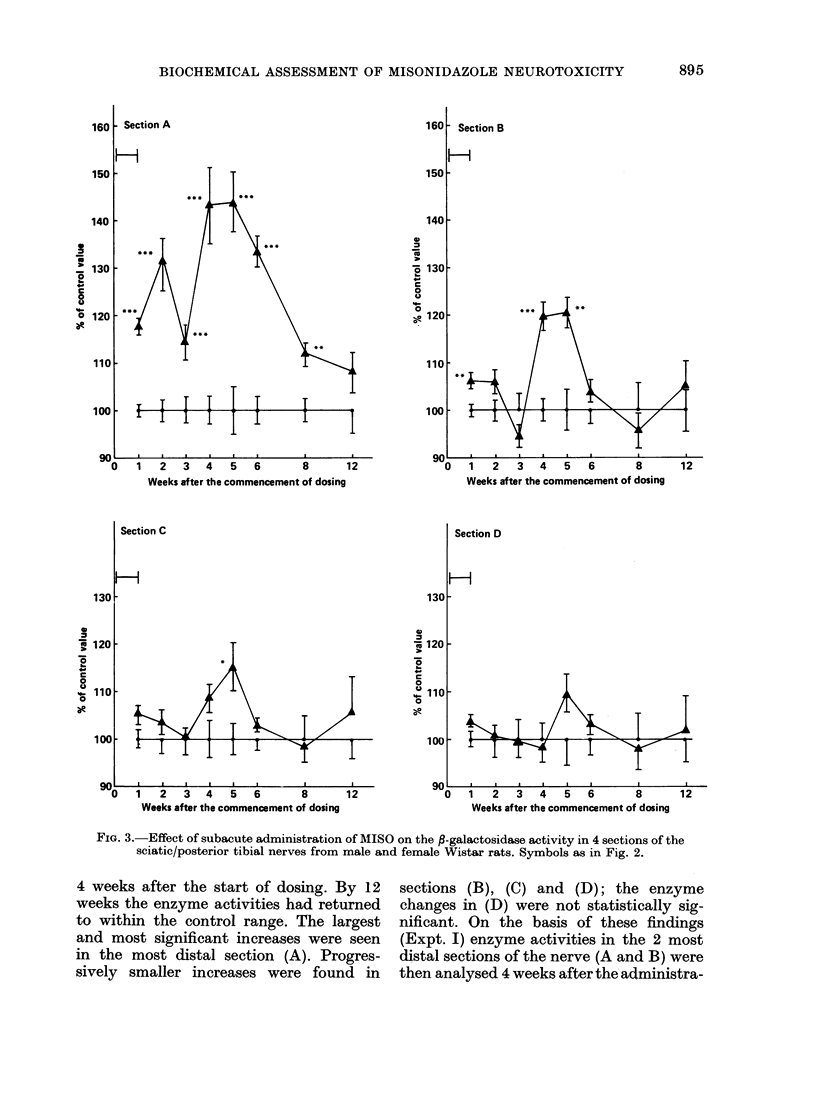

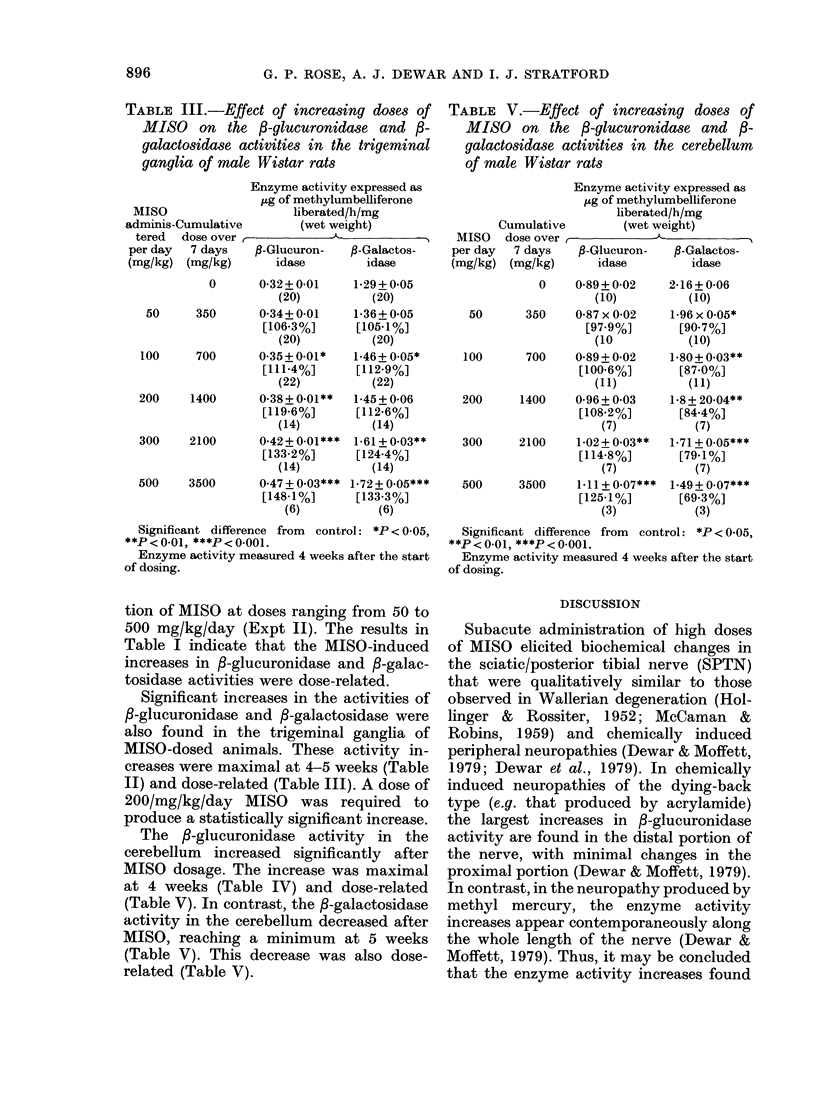

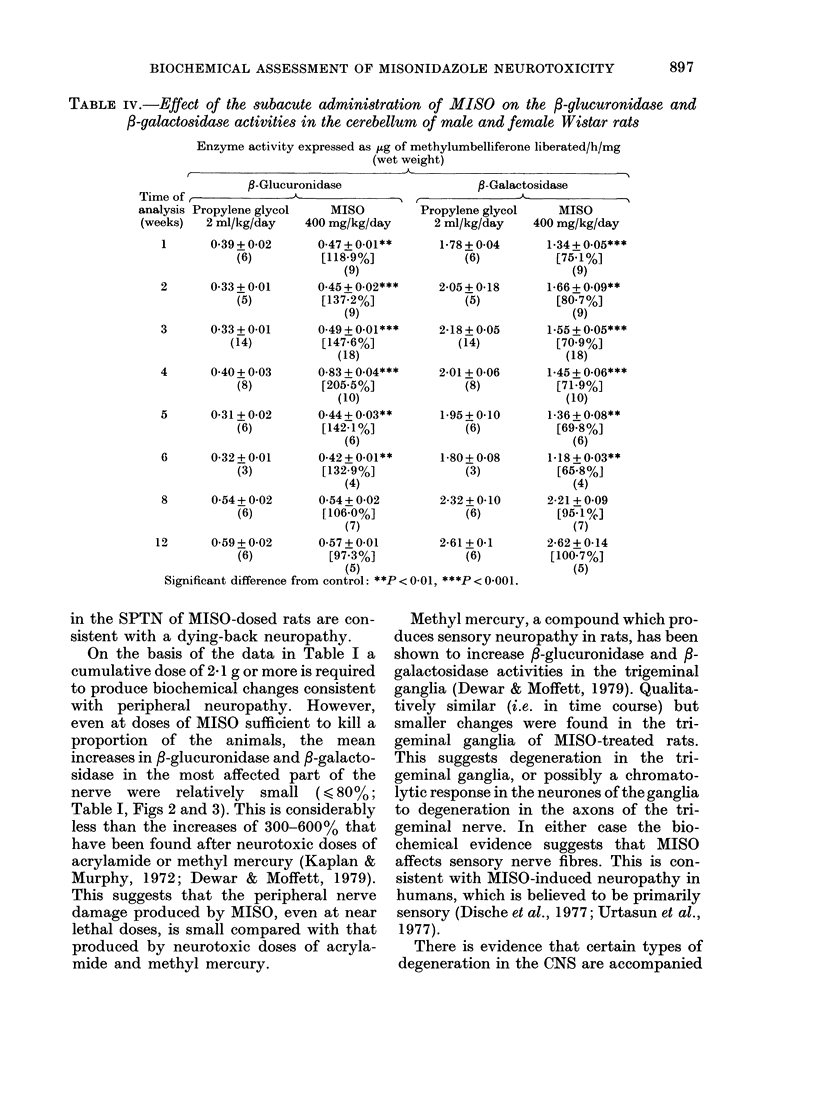

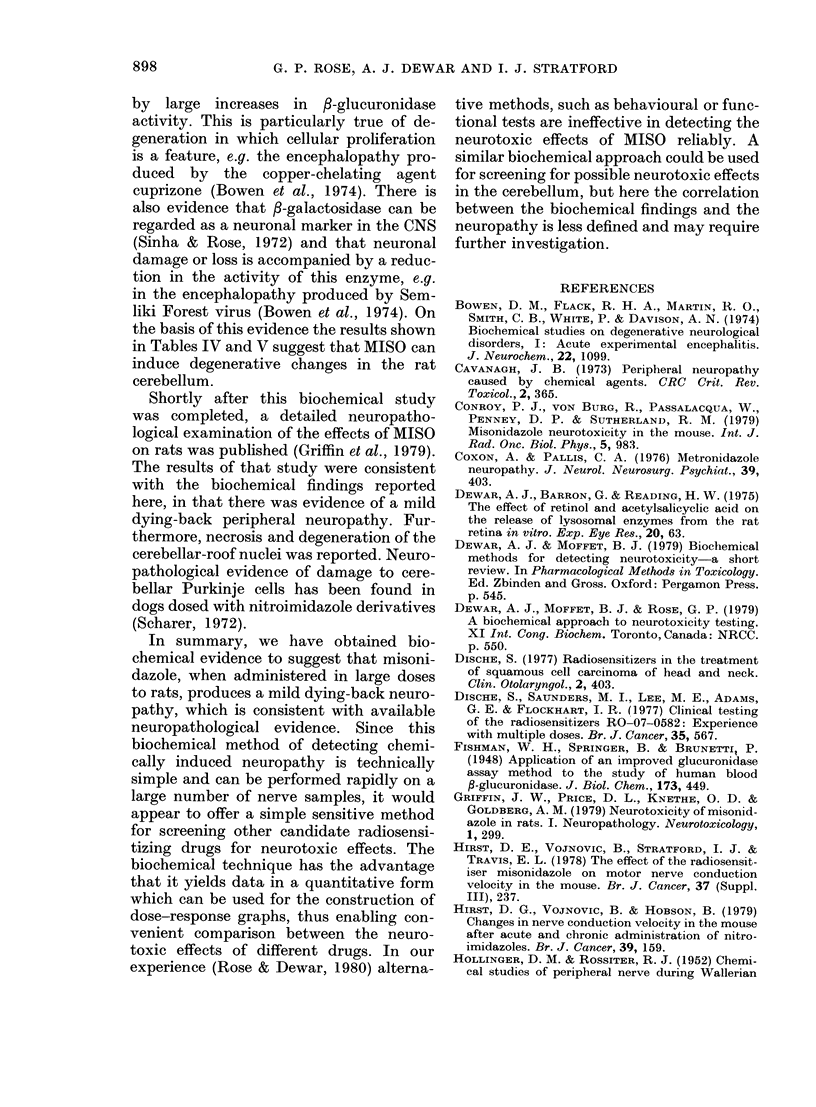

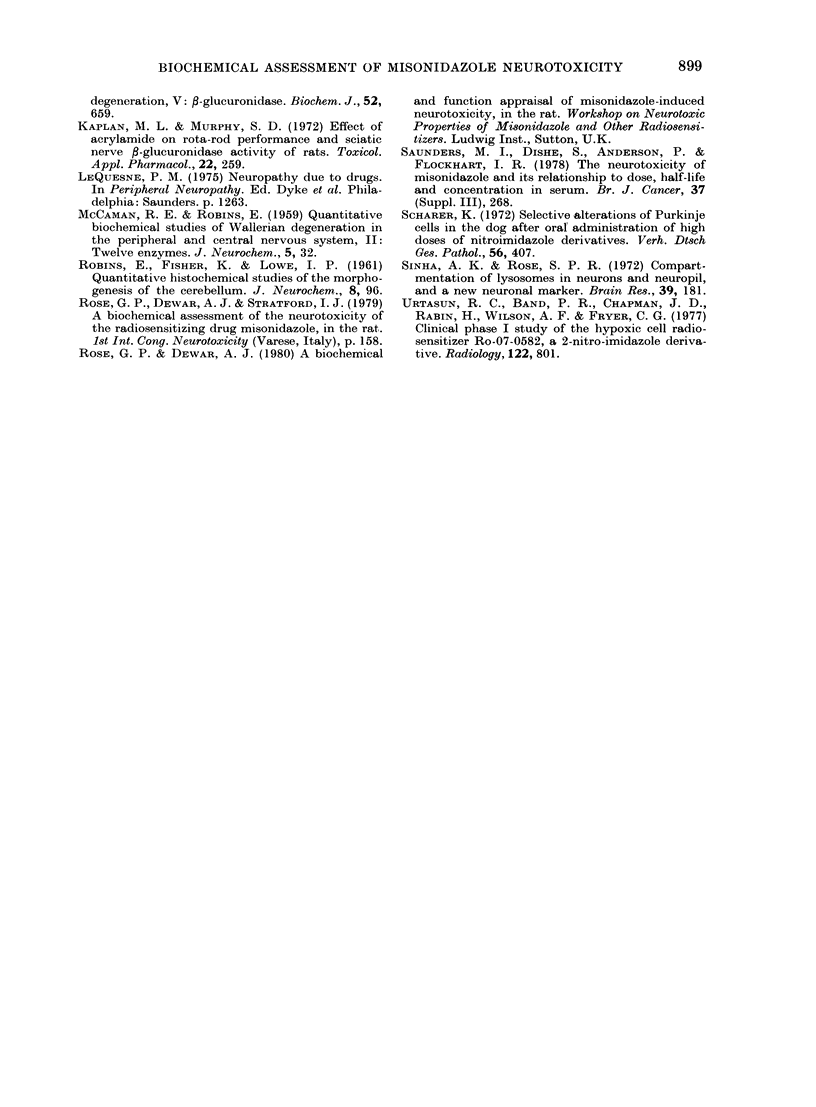


## References

[OCR_01303] Bowen D. M., Flack R. H., Martin R. O., Smith C. B., White P., Davison A. N. (1974). Biochemical studies on degenerative neurological disorders. I. Acute experimental encephalitis.. J Neurochem.

[OCR_01310] Cavanagh J. B. (1973). Peripheral neuropathy caused by chemical agents.. CRC Crit Rev Toxicol.

[OCR_01315] Conroy P. J., Von Burg R., Passalacqua W., Penney D. P., Sutherland R. M. (1979). Misonidazole neurotoxicity in the mouse: evaluation of functional, pharmacokinetic, electrophysiologic and morphologic parameters.. Int J Radiat Oncol Biol Phys.

[OCR_01321] Coxon A., Pallis C. A. (1976). Metronidazole neuropathy.. J Neurol Neurosurg Psychiatry.

[OCR_01326] Dewar A. J., Barron G., Reading H. W. (1975). The effect of retinol and acetylsalicylic acid on the release of lysosomal enzymes from rat retina in vitro.. Exp Eye Res.

[OCR_01345] Dische S. (1977). Radiosensitizers in the treatment of squamous cell carcinoma of the head and neck.. Clin Otolaryngol Allied Sci.

[OCR_01350] Dische S., Saunders M. I., Lee M. E., Adams G. E., Flockhart I. R. (1977). Clinical testing of the radiosensitizer Ro 07-0582: experience with multiple doses.. Br J Cancer.

[OCR_01381] HOLLINGER D. M., ROSSITER R. J. (1952). Chemical studies of peripheral nerve during wallerian degeneration. V. B-Glucuronidase.. Biochem J.

[OCR_01375] Hirst D. G., Vojnovic B., Hobson B. (1979). Changes in nerve conduction velocity in the mouse after acute and chronic administration of nitroimidazoles.. Br J Cancer.

[OCR_01368] Hirst D. G., Vojnovic B., Stratford I. J., Travis E. L. (1978). The effect of the radiosensitizer misonidazole on motor nerve conduction velocity in the mouse.. Br J Cancer Suppl.

[OCR_01390] Kaplan M. L., Murphy S. D. (1972). Effect of acrylamide on rotarod performance and sciatic nerve -glucuronidase activity of rats.. Toxicol Appl Pharmacol.

[OCR_01407] ROBINS E., FISHER H. K., LOWE I. P. (1961). Quantitative histochemical studies of the morphogenesis of the cerebellum. II. Two beta-glycosidases.. J Neurochem.

[OCR_01423] Saunders M. E., Dische S., Anderson P., Flockhart I. R. (1978). The neurotoxicity of misonidazole and its relationship to dose, half-life and concentration in the serum.. Br J Cancer Suppl.

[OCR_01430] Schärer K. (1972). Selektive Purkinje-Zellschädingungen nach oraler Verabreichung gosser Dosen von Nitroimidazol-Derivaten am Hund.. Verh Dtsch Ges Pathol.

[OCR_01436] Sinha A. K., Rose S. P. (1972). Compartmentation of lysosomes in neurones and neuropil and a new neuronal marker.. Brain Res.

[OCR_01440] Urtasun R. C., Band P., Chapman J. D., Rabin H. R., Wilson A. F., Fryer C. G. (1977). Clinical phase I study of the hypoxic cell radiosensitizer RO-07-0582, a 2-nitroimidazole derivative.. Radiology.

